# CED: a conformational epitope database

**DOI:** 10.1186/1471-2172-7-7

**Published:** 2006-04-07

**Authors:** Jian Huang, Wataru Honda

**Affiliations:** 1 Bioinformatics Center, Institute for Chemical Research, Kyoto University, Uji, Kyoto 611-0011, Japan; 2 School of Life Science and Technology, University of Electronic Science and Technology of China, China

## Abstract

**Background:**

Antigen epitopes provide valuable information useful for disease prevention, diagnosis, and treatment. Recently, more and more databases focusing on different types of epitopes have become available. Conformational epitopes are an important form of epitope formed by residues that are sequentially discontinuous but close together in three-dimensional space. These epitopes have implicit structural information, making them attractive for both theoretical and applied biomedical research. However, most existing databases focus on linear rather than conformational epitopes.

**Description:**

We describe CED, a special database of well defined conformational epitopes. CED provides a collection of conformational epitopes and related information including the residue make up and location of the epitope, the immunological property of the epitope, the source antigen and corresponding antibody of the epitope. All entries in this database are manually curated from articles published in peer review journals. The database can be browsed or searched through a user-friendly web interface. Most epitopes in CED can also be viewed interactively in the context of their 3D structures. In addition, the entries are also hyperlinked to various databases such as Swiss-Prot, PDB, KEGG and PubMed, providing wide background information.

**Conclusion:**

A conformational epitope database called CED has been developed as an information resource for investigators involved in both theoretical and applied immunology research. It complements other existing specialised epitope databases. The database is freely available at

## Background

Immune cells recognize epitopes (antigenic determinants) rather than entire antigens. Epitopes are the regions of an antigen that are bound by antigen-specific membrane receptors on lymphocytes or to secreted antibodies[1]. T-cells recognize T-cell epitopes, which are usually linear peptides derived from protein antigens and presented by MHC molecules. B-cells and antibodies recognize B-cell epitopes, which can be complete, small chemical compounds or components of larger macromolecules such as nucleotides, lipids, glycans and proteins. Epitopes from macromolecules, especially proteins, are further classified into another two categories. The first, termed linear epitopes, are segments composed of a continuous string of residues along the polymer chain. The second, termed conformational epitopes, are constituted by several sequentially discontinuous segments that are brought together by the folding of the antigen into its native structure [1,2].

As the molecular basis of immune recognition and the immune response, both kinds of epitope provide valuable information that is useful for disease prevention [3], diagnosis[4,5], and treatment [6-10]. Many databases, focusing on different kinds of epitopes, are available as a large number of epitopes have been identified in the past 20 years. MHCPEP [11], SYFPEITHI[12], FIMM[13], MHCBN[14], EPIMHC[15] are T cell oriented. Bcipep[16] and Epitome[17] are B cell oriented. AntiJen[18] is a multifaceted database with entries on both T cell and B cell epitopes. However, most existing databases focus on linear rather than conformational epitopes.

Despite this, as many as 90% of B-cell epitopes from native proteins are conformational rather than linear [19-22]. In one report, 10 monoclonal antibodies were produced against Helicobacter pylori vacuolating cytotoxin, and all of them proved to react with conformational epitopes [22]. As well as being very common, conformational epitopes may also have functional advantages over linear epitopes. For example, human antibodies directed to conformational epitopes neutralized a wider range of human immunodeficiency virus isolates than human antibodies directed to linear epitopes[23]. A better understanding of conformational epitopes can not only provide useful information for new vaccine design [3] and new diagnostic reagent development[5], but also will be of great value in disease treatment, including against virus infection and cancer [6-10]. Conformational epitopes also have implicit structural information relating to the antigen itself and the mode of binding, which make them attractive to theoretical biology research.

It is money and labour intensive to identify a B cell epitope in detail experimentally. To predict B cell epitopes accurately based on sequence profiling has been a long term goal of many groups. However, a recent report by Blythe et al shows that even the best set of scales and parameters performed only marginally better than random[24]. This suggests that structural approaches and conformational epitope prediction is vital. A conformational epitope prediction server became available recently[25] which will help in conformational epitope discovery, but to predict conformational epitopes accurately is still a very difficult task. For this reason, only several hundred conformational epitopes are currently well defined. We would expect this number to expand rapidly in the future and so it will be necessary to have a database to store and manage all the experimentally well-defined conformational epitopes. Such a specialised conformational epitope database will also be helpful for B cell epitope prediction research.

In this paper, we describe the Conformational Epitope Database (CED). It provides a curated dataset that can be used to evaluate existing epitope prediction methods as well as develop new and better algorithms for prediction. It also complements other existing epitope databases and provides a resource for applied biomedical research in disease prevention, diagnosis, and treatment.

## Construction and content

The MySQL relational database management system is used in CED to store, retrieve and manage the data. The web interface between the user and CED are coded in PHP with PEAR database abstraction layer support. All entries in this database are sourced from articles published in peer reviewed journals. Initially, exhaustive queries are made to PubMed and ScienceDirect; returning more than 3000 references that are loaded into an EndNote reference database. The references are then filtered manually to exclude articles that do not define a conformational epitope or where the defined epitope is only at a very low resolution or completeness. The remaining data is checked and entered manually into CED.

Each entry in CED provides information about a given conformational epitope. The information provided includes:

(1) The residue constitution and location of the epitope

(2) The immunological property of the epitope

(3) The antibody that can bind to the epitope

(4) The experimental method used to identify the epitope

(5) The source antigen where the epitope exists

(6) The sequence of the source antigen

(7) The structure of the source antigen

(8) The structure of the antigen-antibody complex

(9) The chemical properties of the source antigen

(10) The references describing the epitope

(11) The comments and miscellaneous information

**Figure 1:**
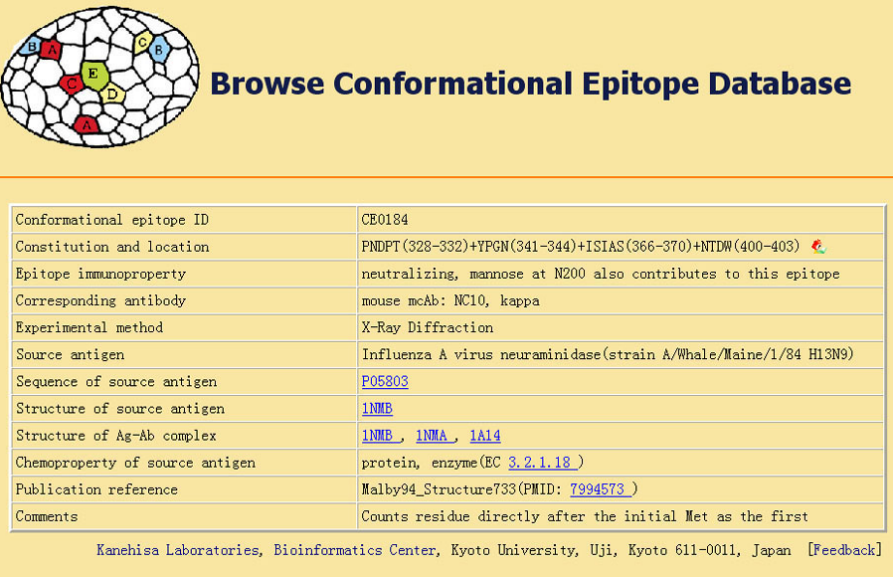
An example entry of CED.

The full description of the entry fields is given Table 1. An example entry is shown in Figure 1.

**Table 1:** Contents of CED database entries

**Entry Field**	**Description**
Conformational epitope ID	Unique identification of the epitope used in CED database.
Constitution and location	Residues that constitute the epitope and their locations.
Epitope immunoproperty	Immunological property of the epitope. For example, any epitope will be considered as neutralizing if the activity of its source antigen is blocked when binding to its corresponding antibody.
Corresponding antibody	Antibody that recognizes and binds this conformational epitope.
Experimental method	Experiment techniques used to identify this epitope, e.g. NMR.
Source antigen	The name of the antigen on which the conformational epitope exists.
Sequence of source antigen	Sequence ID of the source antigen in primary sequence databases such as SwissProt and GenBank; hyperlinked.
Structure of source antigen	PDB ID of the source antigen; hyperlinked
Structure of Ag-Ab complex	PDB ID of the source antigen-antibody complex; hyperlinked.
Chemoproperty of source antigen	Chemical property of source antigen. If the source antigen is an enzyme, it is hyperlinked to KEGG enzyme.
Publication reference	PubMed ID of article that report this epitope; hyperlinked.
Comments	Miscellaneous information of the epitope. It is often about the location difference between the reference and the sequence database.

Currently, CED has 225 entries. The majority (213) are epitopes from protein antigens, though the database also includes 6 from nucleic acids, 5 from glycans and 1 from lipid. 138 epitopes are from vertebrates, the majority from human (109), the rest of the epitopes are from viruses (54), bacteria (22), invertebrates (7), plants (2) and unspecified (2).

## Utility and discussion

### Browsing epitopes in the CED

From the homepage of CED, users can find an introduction to conformational epitopes and CED. They can browse epitope records page by page and entry by entry. When browsing CED, the entries appear in a summary table at first. Only the conformational epitope ID and the constitution and location field are shown, and the data is ordered by the CED identification number (ID). Clicking on an ID opens a new window that displays the information for the selected epitope in detail. An example is shown in Figure 1.

### Searching epitopes in the CED

Users can also search CED for specific conformational epitopes. A detailed help page for the search function is provided. CED can be searched by any field in the entry and by any combination of up to three of the fields. There are three pulldown listboxes and two pairs of radio buttons on the search interface. A query is formed by selecting one or more fields from the pulldown listbox and combining them logically by "and" or "or" radio buttons. Key words or strings, such as a partial protein sequence, the name of an antigen, PDB ID of a structure, clone code of a monoclonal antibody, author name or PMID of a reference are entered into the blank text forms. The HTML form parses the criteria into SQL database queries. The initial results returned are formatted into a summary table as described above. Selecting each ID in the summary table opens a new window that displays the detailed information of each epitope.

**Figure 2:**
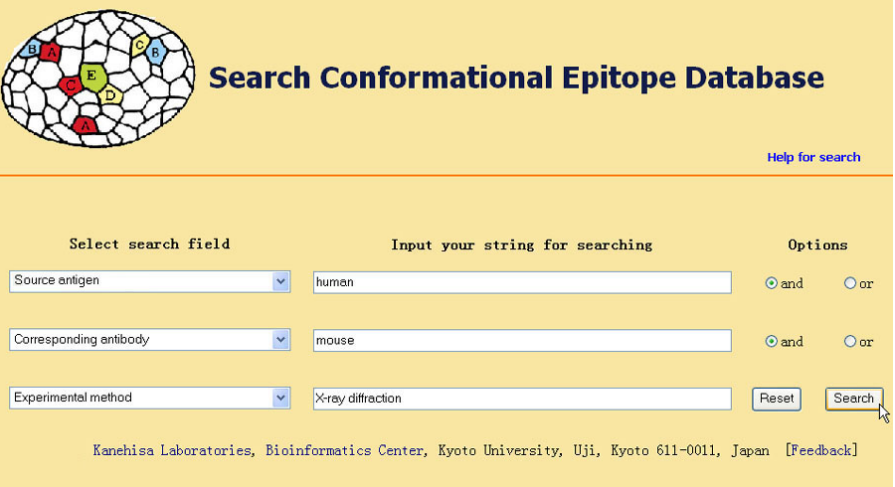
Interface for searching CED and an example search operation.

**Figure 3:**
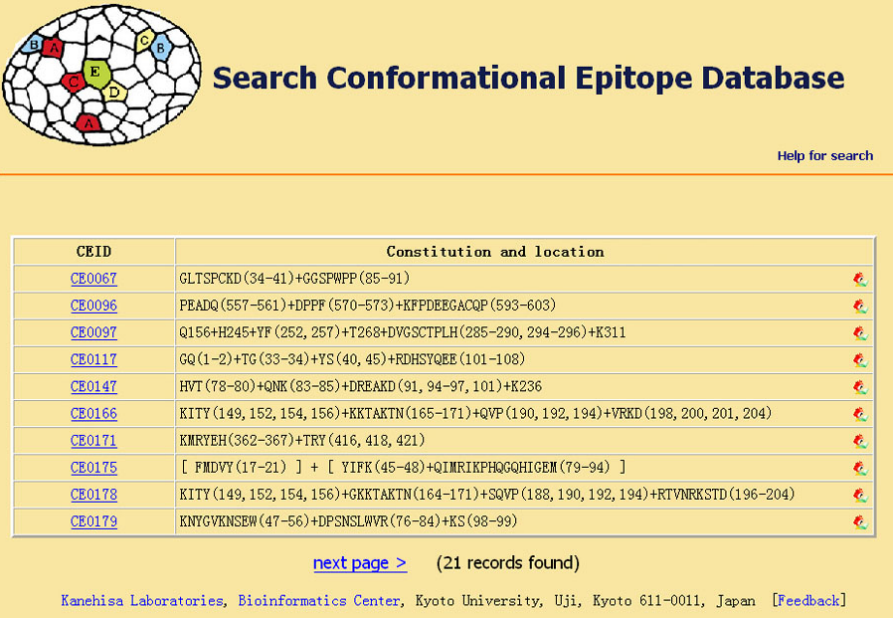
An example search result table.

As an example, if one wanted to retrieve all well defined conformational epitopes from human antigens that are recognized by mouse antibodies and identified by X-ray diffraction, you would first select search field "Source antigen" from the pulldown listbox, input "human" into the corresponding blank text form and then select the "and" button. Then you would select the "Corresponding antibody" search field and input "mouse". Finally, you would select "Experimental method" and input "X-ray diffraction". The operations above will make the search interface appear as shown in Figure 2. After clicking on the "Search" button, records fulfilling the requirements would appear in a new window as shown in Figure 3.

### Viewing epitopes in the CED

**Figure 4:**
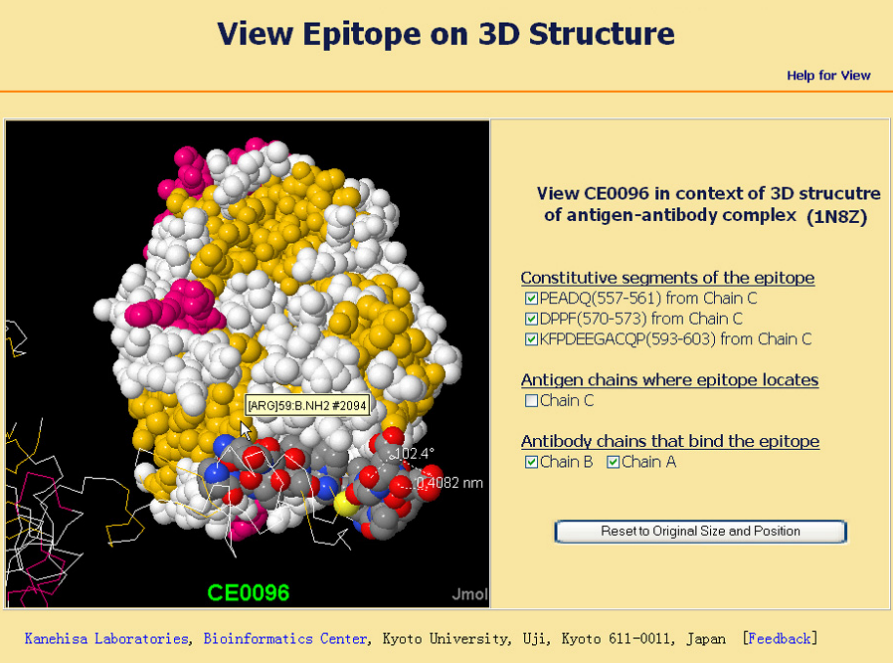
An example visualisation of CE0096. Epitope CE0096 has 3 segments, which are displayed as spacefill colored by CPK; the other part of antigen is visualised as backbone colored by secondary structure. The antibody chains are displayed as spacefill colored by secondary structure.

Most conformational epitopes in CED can be viewed interactively in the context of the antigen-antibody complex, antigen structure or known theoretical model, if they have corresponding PDB structures. The visualization function of CED is powered by Jmol. To view an epitope in the context of its 3D structure can help users identify important structural features and judge if entries defined by non-structural methods are reasonable. When browsing or searching the CED, entries that have PDB structures will have a conspicuous view icon in both the summary table and the entry table. Clicking the view icon will initialize the loading of the Jmol Java applet. By default, structures in CED are displayed as backbone colored by secondary structure. After loading, users can turn on or turn off the epitope segments, antigen chains, and antibody chains. When turned on, epitope segments "blink" and are then displayed in spacefill mode colored by CPK. Users can also zoom in, zoom out, move, spin, and rotate the structure, or even measure distance, angle, and dihedral angle. A help page for viewing epitopes can be found through a link on the view page. An example visualisation of CE0096 is shown in Figure 4.

### Necessity of building a specialised conformational epitope database

Conformational epitopes can not only provide useful information for new vaccine design [3] and new diagnostic reagent development [5], but also will be of great value in disease treatment, including against virus infection and cancer [6-10]. For example, two conformational epitopes in CED (CE0096 and CE0199) are targets of two FDA approved drugs (Herceptin and Erbitux), which are effective in treating some cancers [6,9]. We need a specialised database to store this kind of useful information. Conformational epitopes also provide structural information relating to the antigen itself, making them attractive to theoretical biology research. A recent research clearly shows the underperformance of existing linear B cell epitope prediction methods [24], suggesting that structural approaches and conformational epitope prediction is vital. Thus a specialised conformational epitope database will be helpful for developing new B cell epitope prediction methods.

### Related databases

The existing epitope databases can be classified into four main categories: T cell oriented such as MHCPEP[11], SYFPEITHI[12], FIMM[13], MHCBN[14], EPIMHC[15]; B cell oriented such as Bcipep[16] and Epitome[17]; single pathogenic organism oriented such as the HIV Molecular Immunology Database [26] and HCV Molecular Immunology Database[27] ; multifaceted database such as AntiJen[18] and IEDB[28]. IEDB is still under construction at the time of writing.

Compared with most existing epitope databases, the current size of CED is limited. However, most existing epitope databases are focused on collecting linear epitopes; whereas CED only contains conformational epitopes, which are often less well defined and are harder to identify experimentally or theoretically. Many articles report new conformational epitopes, but few are defined completely and precisely enough for inclusion in CED. Taking autoimmune epitopes as an example: In many cases, the nature of the epitopes have often been successively defined and refined from the level of whole cellular organelles (using immunofluorescence methods), to identifying the macromolecule involved (immunoblot, gene expression libraries), to epitope regions (truncated cDNAs, peptide scanning), but few are identified at the contact residue level [29]. Since data quality is vital in bioinformatics research, the aim of CED is to provide high quality, well defined conformational epitope information. So conformational epitopes that are not defined clearly are discarded which limits the database size.

Epitome[17], a very recently released B cell epitope database has a similar size to CED. Epitome collects B cell epitopes only from PDB structures and includes CDR information. In contrast, CED is sourced from the literature and also has conformational epitopes defined by methods other than X-ray diffraction and NMR, such as scanning mutagenesis, overlapping peptides, and phage display. Although CED and Epitome are similar they are derived from different resources and so provide complementary information. We believe that a simple database, such as CED, specialising in conformational epitopes has value in conjunction with these more complex, less specialised, databases. Although our manual search procedure may have missed a few known conformational epitopes, we believe that CED is an essentially complete database of well defined conformational epitopes.

### Future work

Firstly, like all other databases, errors will have occurred during the data accumulation phase. We hope users will send their feedback to help us maintain and revise CED in future.

Secondly, we will scan newly published peer review articles for well defined or refined conformational epitopes routinely. New epitopes will be added; and newly refined epitopes will be updated. Due to the rapid progress of techniques in this field, we expect that more and more new conformational epitopes will be identified in the future. Thus, both the quantity and quality of CED entries will increase.

Lastly, we have noticed that a formal ontology of epitopes has been developed and suggested recently[30]. To represent and communicate epitope information systematically and effectively, we will make the next release of CED completely compatible with IEDB's ontology.

## Conclusion

A conformational epitope database (CED) has been developed, which can be browsed or searched through a simple user friendly web interface. It is an essentially complete database of well defined conformational epitopes and provides a complement to other existing specialised epitope databases. CED is also hyperlinked to several external databases, providing wide background information for each entry. Though currently relatively small in size, the data in CED provides valuable information for disease prevention, diagnosis, and treatment. Thus, we hope it will be an important information resource for investigators involved in both theoretical and applied immunology research.

## Availability and requirements

The database is available at , suitable for most graphical web browsers support Java applets and JavaScript. We have tested on the Windows, Mac and Linux operating systems.

## Abbreviations

CED: Conformational Epitope Database; CDR: Complementarity-Determining Region; HCV: Hepatitis C Virus; HIV: Human Immunodeficiency Virus; HTML: Hypertext Markup Language; IEDB: Immune Epitope Database and Analysis Resource; KEGG: Kyoto Encyclopaedia of Genes and Genomes; MHC: Major Histocompatibility Complex; NCBI: National Center for Biotechnology Information; PEAR: PHP Extension and Application Repository; PDB: Protein Data Bank; PHP: Hypertext Preprocessor; SQL: Structured Query Language.

## Authors' contributions

JH and WH extracted the data, compiled the database and wrote the code for the web interface. All authors have read and approved the final manuscript.

## References

[B1] Goldsby RA, Kindt TJ, Kuby J, Osborne BA (2002). Immunology.

[B2] Barlow DJ, Edwards MS, Thornton JM (1986). Continuous and discontinuous protein antigenic determinants. Nature.

[B3] Saxena AK, Singh K, Su HP, Klein MM, Stowers AW, Saul AJ, Long CA, Garboczi DN (2005). The essential mosquito-stage P25 and P28 proteins from Plasmodium form tile-like triangular prisms. Nat Struct Mol Biol.

[B4] Matsuo H, Kohno K, Niihara H, Morita E (2005). Specific IgE Determination to Epitope Peptides of {omega}-5 Gliadin and High Molecular Weight Glutenin Subunit Is a Useful Tool for Diagnosis of Wheat-Dependent Exercise-Induced Anaphylaxis. J Immunol.

[B5] Tegoni M, Spinelli S, Verhoeyen M, Davis P, Cambillau C (1999). Crystal structure of a ternary complex between human chorionic gonadotropin (hCG) and two Fv fragments specific for the alpha and beta-subunits. J Mol Biol.

[B6] Li S, Schmitz KR, Jeffrey PD, Wiltzius JJ, Kussie P, Ferguson KM (2005). Structural basis for inhibition of the epidermal growth factor receptor by cetuximab. Cancer Cell.

[B7] Nybakken GE, Oliphant T, Johnson S, Burke S, Diamond MS, Fremont DH (2005). Structural basis of West Nile virus neutralization by a therapeutic antibody. Nature.

[B8] Adams GP, Weiner LM (2005). Monoclonal antibody therapy of cancer. Nat Biotechnol.

[B9] Cho HS, Mason K, Ramyar KX, Stanley AM, Gabelli SB, Denney DW, Leahy DJ (2003). Structure of the extracellular region of HER2 alone and in complex with the Herceptin Fab. Nature.

[B10] Riemer AB, Kurz H, Klinger M, Scheiner O, Zielinski CC, Jensen-Jarolim E (2005). Vaccination with cetuximab mimotopes and biological properties of induced anti-epidermal growth factor receptor antibodies. J Natl Cancer Inst.

[B11] Brusic V, Rudy G, Harrison LC (1998). MHCPEP, a database of MHC-binding peptides: update 1997. Nucleic Acids Res.

[B12] Rammensee H, Bachmann J, Emmerich NP, Bachor OA, Stevanovic S (1999). SYFPEITHI: database for MHC ligands and peptide motifs. Immunogenetics.

[B13] Schonbach C, Koh JL, Flower DR, Brusic V (2005). An update on the functional molecular immunology (FIMM) database. Appl Bioinformatics.

[B14] Bhasin M, Singh H, Raghava GP (2003). MHCBN: a comprehensive database of MHC binding and non-binding peptides. Bioinformatics.

[B15] Reche PA, Zhang H, Glutting JP, Reinherz EL (2005). EPIMHC: a curated database of MHC-binding peptides for customized computational vaccinology. Bioinformatics.

[B16] Saha S, Bhasin M, Raghava GP (2005). Bcipep: a database of B-cell epitopes. BMC Genomics.

[B17] Schlessinger A, Ofran Y, Yachdav G, Rost B (2006). Epitome: database of structure-inferred antigenic epitopes. Nucleic Acids Res.

[B18] Toseland CP, Clayton DJ, McSparron H, Hemsley SL, Blythe MJ, Paine K, Doytchinova IA, Guan P, Hattotuwagama CK, Flower DR (2005). AntiJen: a quantitative immunology database integrating functional, thermodynamic, kinetic, biophysical, and cellular data. Immunome Res.

[B19] Horsfall AC, Hay FC, Soltys AJ, Jones MG (1991). Epitope mapping. Immunol Today.

[B20] Padlan EA (1996). X-ray crystallography of antibodies. Adv Protein Chem.

[B21] Benjamin DC (1995). B-cell epitopes: fact and fiction. Adv Exp Med Biol.

[B22] Vinion-Dubiel AD, McClain MS, Cao P, Mernaugh RL, Cover TL (2001). Antigenic diversity among Helicobacter pylori vacuolating toxins. Infect Immun.

[B23] Steimer KS, Scandella CJ, Skiles PV, Haigwood NL (1991). Neutralization of divergent HIV-1 isolates by conformation-dependent human antibodies to Gp120. Science.

[B24] Blythe MJ, Flower DR (2005). Benchmarking B cell epitope prediction: underperformance of existing methods. Protein Sci.

[B25] Kulkarni-Kale U, Bhosle S, Kolaskar AS (2005). CEP: a conformational epitope prediction server. Nucleic Acids Res.

[B26] HIV Molecular Immunology Database. http://www.hiv.lanl.gov/content/immunology/index.html.

[B27] Yusim K, Richardson R, Tao N, Dalwani A, Agrawal A, Szinger J, Funkhouser R, Korber B, Kuiken C (2005). Los alamos hepatitis C immunology database. Appl Bioinformatics.

[B28] Peters B, Sidney J, Bourne P, Bui HH, Buus S, Doh G, Fleri W, Kronenberg M, Kubo R, Lund O, Nemazee D, Ponomarenko JV, Sathiamurthy M, Schoenberger SP, Stewart S, Surko P, Way S, Wilson S, Sette A (2005). The design and implementation of the immune epitope database and analysis resource. Immunogenetics.

[B29] Mackay IR, Rowley MJ (2004). Autoimmune epitopes: autoepitopes. Autoimmun Rev.

[B30] Sathiamurthy M, Peters B, Bui HH, Sidney J, Mokili J, Wilson SS, Fleri W, McGuinness DL, Bourne PE, Sette A (2005). An ontology for immune epitopes: application to the design of a broad scope database of immune reactivities. Immunome Res.

